# Multi-Scale Imaging of Vascular Pathologies in Cardiovascular Disease

**DOI:** 10.3389/fmed.2021.754369

**Published:** 2022-01-05

**Authors:** Ashish Tiwari, Betsalel Elgrably, Galit Saar, Katrien Vandoorne

**Affiliations:** ^1^Faculty of Biomedical Engineering, Technion-Israel Institute of Technology, Haifa, Israel; ^2^Biomedical Core Facility, Rappaport Faculty of Medicine, Technion-Israel Institute of Technology, Haifa, Israel

**Keywords:** endothelial cells, cardiovascular disease, multimodality imaging, optical imaging, positron emission tomography, magnetic resonance imaging, single photon emission contrast tomography

## Abstract

Cardiovascular disease entails systemic changes in the vasculature. The endothelial cells lining the blood vessels are crucial in the pathogenesis of cardiovascular disease. Healthy endothelial cells direct the blood flow to tissues as vasodilators and act as the systemic interface between the blood and tissues, supplying nutrients for vital organs, and regulating the smooth traffic of leukocytes into tissues. In cardiovascular diseases, when inflammation is sensed, endothelial cells adjust to the local or systemic inflammatory state. As the inflamed vasculature adjusts, changes in the endothelial cells lead to endothelial dysfunction, altered blood flow and permeability, expression of adhesion molecules, vessel wall inflammation, thrombosis, angiogenic processes, and extracellular matrix production at the endothelial cell level. Preclinical multi-scale imaging of these endothelial changes using optical, acoustic, nuclear, MRI, and multimodal techniques has progressed, due to technical advances and enhanced biological understanding on the interaction between immune and endothelial cells. While this review highlights biological processes that are related to changes in the cardiac vasculature during cardiovascular diseases, it also summarizes state-of-the-art vascular imaging techniques. The advantages and disadvantages of the different imaging techniques are highlighted, as well as their principles, methodologies, and preclinical and clinical applications with potential future directions. These multi-scale approaches of vascular imaging carry great potential to further expand our understanding of basic vascular biology, to enable early diagnosis of vascular changes and to provide sensitive diagnostic imaging techniques in the management of cardiovascular disease.

## Introduction

Cardiovascular disease (CVD) is the leading cause of death worldwide and is associated with several chronic diseases and adverse health outcomes. A multitude of etiologies can jeopardize heart function, and subsequently lead to heart failure. Some well-recognized etiological factors include lack of exercise, diabetes, unhealthy diet, smoking and family history (1). In CVDs, inflammation activates the endothelial cells lining the systemic vasculature (2), involving a complex interplay between molecular mediators, endothelial cells and immune cells. Systemic changes to the endothelium constitute key early events that shape CVDs through their direct impact on the heart and large vessels and are a hallmark of CVD progression and pathogenesis. For instance, a systemically inflamed endothelium initiates atherosclerotic plaques, which can remain asymptomatic for decades, until a plaque rupture narrows or occludes a vessel (2). Occlusion of vessels impedes blood flow to vital organs, such as the heart, causing myocardial infarction (MI). Identifying the mechanisms leading to atherosclerosis and MI, has the potential to distinctly lower cardiovascular mortality. Therefore, an in-depth understanding, combined with early detection and prognosis of endothelial changes hold the potential to prevent fatal events by enabling early treatment, minimizing the risk and reducing severity of CVD. This review covers imaging techniques used to detect early and late vascular changes in the heart and the large vessels during CVDs.

### Vascular Biology

**Figure 1:**
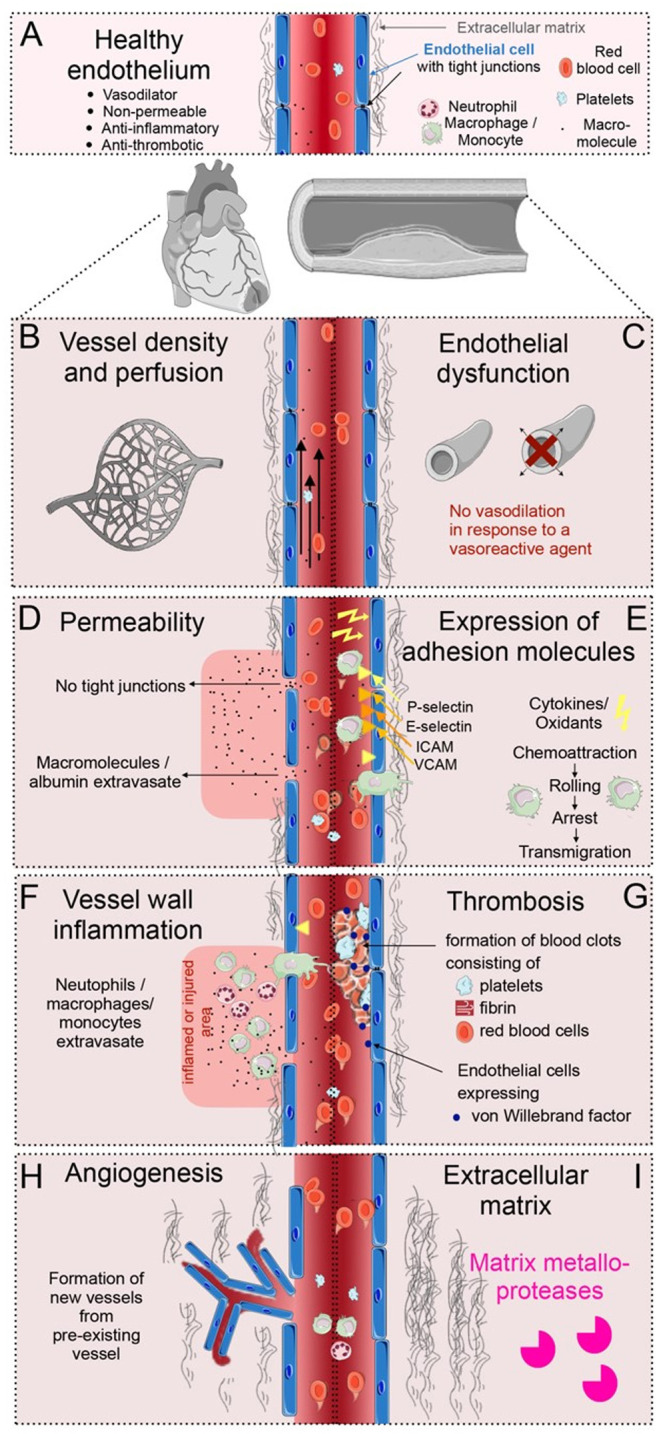
Overview of vascular processes in cardiovascular disease (CVD). **(A)** The healthy endothelium of the heart and the large vessels, is able to dilate, is non-permeable, anti-inflammatory and anti-thrombotic, with tight junction between the endothelial cells, and a balanced amount of extracellular matrix (ECM) surrounding the vessels. **(B)** Alterations in the vessel density and perfusion occur during CVD. **(C)** Endothelial dysfunction is a condition that occurs early in the pathogenesis of CVD, debilitating the local vasodilator response to vasoactive agents. **(D)** Vascular permeability occurs when tight junctions between endothelial cells loosen; molecules and proteins can then extravasate from the circulation. **(E)** Adhesion molecules occurring in specific regions of the vasculature slow down leukocytes at specific regions of the endothelium. **(F)** Vessel wall inflammation occurs when neutrophils, monocytes and macrophages extravasate into an inflamed or injured area. **(G)** Thrombosis is induced when activated endothelial cells release thrombotic factors, e.g., von Willebrand factor, and form blood clots at the vascular wall together with fibrin, platelets and red blood cells. **(H)** Angiogenesis is the formation of new blood vessels. This happens during infarct healing and in atherosclerotic plaque development. **(I)** Irregular ECM turnover in CVD is a product of imbalanced ECM production and proteolytic activities by matrix metalloproteases.

The vascular system transports nutrients and oxygen to all organs and enables the circulation of blood and immune cells throughout the body. The endothelium, first identified by Wilhelm His in 1865, comprises the inner cell layer of blood vessels and lymphatics and is present in all adult mammalian tissues. The introduction of electron microscopy (EM) in the 1960's, supplied new insights into and a better understanding of the tightly connected endothelium comprised of the endothelial cells surrounded by a continuous basement membrane (3). During steady state, the vessel density and perfusion of the heart contribute to the normal function of the heart. A healthy endothelium is considered a regulator of vascular tones; in response to vasoactive agents, the normal-functioning endothelium vasodilates as it produces nitric oxide (NO) (4, 5). Cell junctions between endothelial cells are tight at the endothelium of the heart and large vessels, to prevent leakage of plasma proteins in these vital areas (2, 3). Moreover, under normal circumstances the endothelium is anti-inflammatory, as it resists prolonged contact with circulating leukocytes (2). The steady-state endothelium is anti-thrombotic, inhibiting the formation of blood clots in the vasculature (6). In addition, in healthy adult heart and large vessels, the formation of new vessels from preexisting vessels, called angiogenesis is imperceptible. Finally, during homeostasis, endothelial cells are surrounded by a balanced amount of extracellular matrix (ECM) supporting the vasculature (3, 7, 8). All these features of healthy endothelial cells are important for cardiovascular health (Figure 1A).

In the pathogenesis of CVDs, endothelial cells undergo modifications as the vasculature adjusts to the inflammatory state (2). Systematic vascular changes are crucial for prediction of the early endothelial pathologies that can be imaged during CVD progression. Endothelial cells interact with circulating innate immune cells supplied by hematopoietic organs in the body, and therefore play a crucial role in the development and eventual outcome of CVD. Non-invasive imaging techniques can be used as a translational tool to study and quantify endothelial cell features and changes (9). Endothelial cell imaging and monitoring of vascular changes can provide improved strategies for early detection and risk stratification in CVDs.

In cardiomyopathies, reduced perfusion or even occlusion of vessels induces ischemia in the heart (Figure 1B) (7). Early in the pathogenesis of CVDs, the endothelial vasomotor function is compromised resulting in an impaired arterial vasoreactivity (i.e., a diminished vasodilator response to vasoactive agents) (Figure 1C) (4, 5). In addition, early in CVD development, the endothelial cells can become permeable as tight junctions loosen. Permeable vessels allow for extravasation of serum proteins in cardiac and plaque-associated vasculature (Figure 1D) (2, 3). Furthermore, during inflammatory conditions, endothelial cells express adhesion molecules, which slow down circulating leukocytes (Figure 1E). When these leukocytes cross the vessel wall, transmigrating neutrophils, macrophages and monocytes cause vessel wall inflammation, breaching the vessel wall, and releasing cytokines (Figure 1F) (6). Additionally, activated endothelial cells can release procoagulant and prothrombotic factors, subsequently inducing blood clots in the circulation (Figure 1G) (10). Moreover, proangiogenic programs in endothelial cells can be activated during CVDs, inducing the development of new vessels, specifically during infarct healing and in atherosclerotic plaque development (Figure 1H) (3, 7). Finally, the unbalanced rearrangement of the ECM and activation of proteolytic activities surrounding the vasculature are crucial biological processes in the progression and outcome of CVDs (Figure 1I) (8). Although, there are distinct biological processes that occur at different stages of CVD progression, some, such as endothelial dysfunction, vascular permeability, and perfusion defects can co-occur or overlap. These endothelial changes take place in a complex microenvironment, involving interaction with many other cell types, such as leukocytes, perivascular smooth muscle cells, fibroblasts and the extracellular matrix.

### Multi-Scale Imaging Modalities in Vascular Imaging

Current molecular imaging techniques of the heart and the large vessels allow for new biological understanding, early detection and diagnosis of CVDs, as well as targeted treatments and therapy monitoring. Key vascular information can be obtained *via* molecular multi-scale imaging, ranging from optical micro-scale imaging, optoacoustic imaging, and ultrasound, to macro-scale single-photon emission computed tomography (SPECT), positron emission tomography (PET), X-ray computed tomography (CT), and magnetic resonance imaging (MRI) imaging (Table 1).

**Table 1:** Multi-scale imaging modalities and their technical specifications.

**Imaging modality**	**Spatial resolution**	**Depth of penetration**	**Scan time**	**Sensitivity (mol/L)**	**Molecular probes**
IVM	1–10 μm	<700 μm	Sec-hrs	Single cell	Fluorescent, near-infrared dyes
Optical fluorescence	2–3 mm	<1 mm	Sec-min	10^−9^-10^−12^	Fluorescent, near-infrared dyes
Optical bioluminescence	3–5 mm	1–2 cm	Sec-min	10^−15^-10^−17^	Luciferase
Optoacoustic imaging	10 μm−1 mm	mm-cm	Sec-min	N/A	Endogenous, near-infrared dyes
Ultrasound	30–500 μm	mm-cm	Sec-min	10^−6^-10^−9^	Microbubble
SPECT	0.3–1 mm	No limit	Min	10^−10^-10^−11^	^111^In, ^67^Ga, ^99^Tcm
PET	1–2 mm	No limit	Sec-min	10^−11^-10^−12^	^18^F, ^64^Cu, ^68^Ga, ^89^Zr
CT	25–250 μm	No limit	Min	N/A	I, Au
MRI	50–250 μm	No limit	Min-hrs	10^−3^-10^−5^	Gd, Fe, Eu, Mn

*IVM, Intravital microscopy; SPECT, single-photon emission computed tomography; PET, positron emission tomography; CT, X-ray computed tomography; MRI, magnetic resonance imaging*.

Optical imaging includes both high-resolution micro-scale intravital microscopy (IVM) as well as whole-body optical fluorescence and bioluminescence imaging. IVM can visualize cellular and subcellular biological processes at early and late stages of CVD, both *ex vivo* and with high sensitivity *in vivo* (11). While it enables visualization at single-cell resolution, it is often time-consuming and necessitates dedicated experts to run the system (11). One of the disadvantages of optical imaging is the limited penetration depth and the lack of anatomical information, with fluorescence imaging in mice performing as deep as 2–3 cm (12), and bioluminescence imaging up to 5 mm deep (13). Both optical fluorescence and bioluminescence imaging are high-throughput and are applied to validate cellular internalization of various imaging agents and for intracellular tracking of small objects. Both optical imaging techniques are highly sensitive (fluorescence: 10^−9^-10^−12^ M; bioluminescence: 10^−15^-10^−17^ M; Table 1), but their spatial resolution is limited. Due to its relatively low cost and high-throughput, optical imaging is widely used for a broad range of imaging applications (12–14). A range of commercially available fluorescence probes, such as organic fluorescent and near-infrared dyes, and bioluminescence imaging probes, like luciferase, are widely used in vascular imaging application (14).

Unlike other imaging modalities like CT, SPECT, PET, and MRI, both optoacoustic and ultrasound imaging have limited penetration depth (Table 1). Optoacoustic is an alternative optical-based technique that allows for both microscopic and deeper whole-body imaging. In optoacoustic imaging, the target tissue absorbs light and heats up, which is accompanied by tissue expansion. This expansion results in emission of an acoustic signal, which can be detected with an ultrasound transducer. The use of near-infrared pulses of light, together with the absence of acoustic background signal, provides for increased resolution depth. Contrast is present due to endogenous proteins (hemoglobin, melanin), and near-infrared probes (14, 15). Ultrasound imaging, a real-time anatomical imaging modality, is used to visualize the heart and the vasculature in disease diagnosis. Ultrasonic waves are transduced around the tissue and subsequently a backscattered wave is generated. This wave is then recorded, and an image is generated. In preclinical research, the acoustic response of targeted microbubbles is measured to visualize targeted molecular changes in CVD. These microbubbles allow for high sensitivity (10^−9^ M) and accuracy (16–19).

CT, SPECT, PET and MRI are imaging techniques with unlimited penetration depth. CT is not considered to offer high sensitivity, and thus has limited ability to image cells. Iodine (I) or gold (Au) are used to provide contrast or label cells. CT visualizes differences in tissue attenuation of x-rays, but is hardly used for gaining molecular information. PET and SPECT imaging techniques offer very high sensitivity for radiolabeled probes for targeted molecules in the body, however they fail to provide anatomical information. Therefore, they are most often combined with CT. Both PET and SPECT imaging provide quantitative biomarker information and can achieve high sensitivity of 10^−11^ M. SPECT uses radionuclides, such as gamma emitters; emitted gamma rays are detected to create the images. In PET, a radionuclide annihilates an electron and emits two photons that are detected in a coincident fashion. Combined PET/MRI systems is an additional emerging technology that aims to capitalize the advantages of MRI, including increased soft tissue contrast, tissue characterization and molecular imaging, in combination with PET. MRI can image tissue, organ and whole-body anatomy, with unlimited depth of penetration and high sensitivity. gadolinium (Gd), iron (Fe), europium (Eu), and Manganese (Mn) are contrast agents that can produce sufficient contrast in various MRI techniques. While its sensitivity (~10^−4^ M) is lower than modalities such as PET or SPECT (Table 1), its accurate anatomical soft tissue information and versatility (i.e., the availability of multiple imaging strategies like T_1_, T_2_, CEST) render MRI an appreciated molecular imaging technique in CVD. MRI is applied to assess vascular changes, recruitment of immune cells and for dynamic distribution imaging. The main disadvantage of MRI is its temporal resolution, requiring minutes to hours to complete a scan (20). A hurdle in all imaging modalities, that should be noted for cardiovascular imaging, is the motion of the lungs, heart, and large vessels, which is several orders of magnitude larger than the cellular and molecular processes of interest. Therefore, accurate cardiac and respiratory gating, either prospectively or retrospectively, is essential in vascular imaging of CVD (11).

Biomarkers verified on a single-cell level by optical imaging in preclinical CVD animal models can be converted to optoacoustic, ultrasound, SPECT, PET, or MRI markers when bound to specific contrast agents. These molecular targets could be imaged by ultrasound, SPECT, PET, and MRI molecular imaging techniques and advance novel preclinical molecular imaging techniques that eventually could reach the clinics. From a basic science perspective, multi-scale vascular imaging and advances in imaging instrumentation and quantification have enhanced insights into vascular biology and promoted the exploration of novel molecular targets on endothelial cells, which can be applied to verify vascular changes in animal models. Novel vascular imaging techniques can be translated to the clinic for diagnostics, and for monitoring response to treatment.

## Multi-scale Imaging of Heart and Large Vessels

### Vessel Density and Perfusion

Sufficient blood flow and perfusion are vital for normal functioning of the heart. Therefore, reduced cardiac vessel density and perfusion can indicate cardiomyopathies and vessel occlusion suggest ischemic states. Cardiac imaging techniques measuring cardiac capillary network density, myocardial blood flow, perfused capillary blood volume, and first-pass distribution volume are meaningful for assessment of cardiovascular health (Figure 1B) (7).

**Figure 2:**
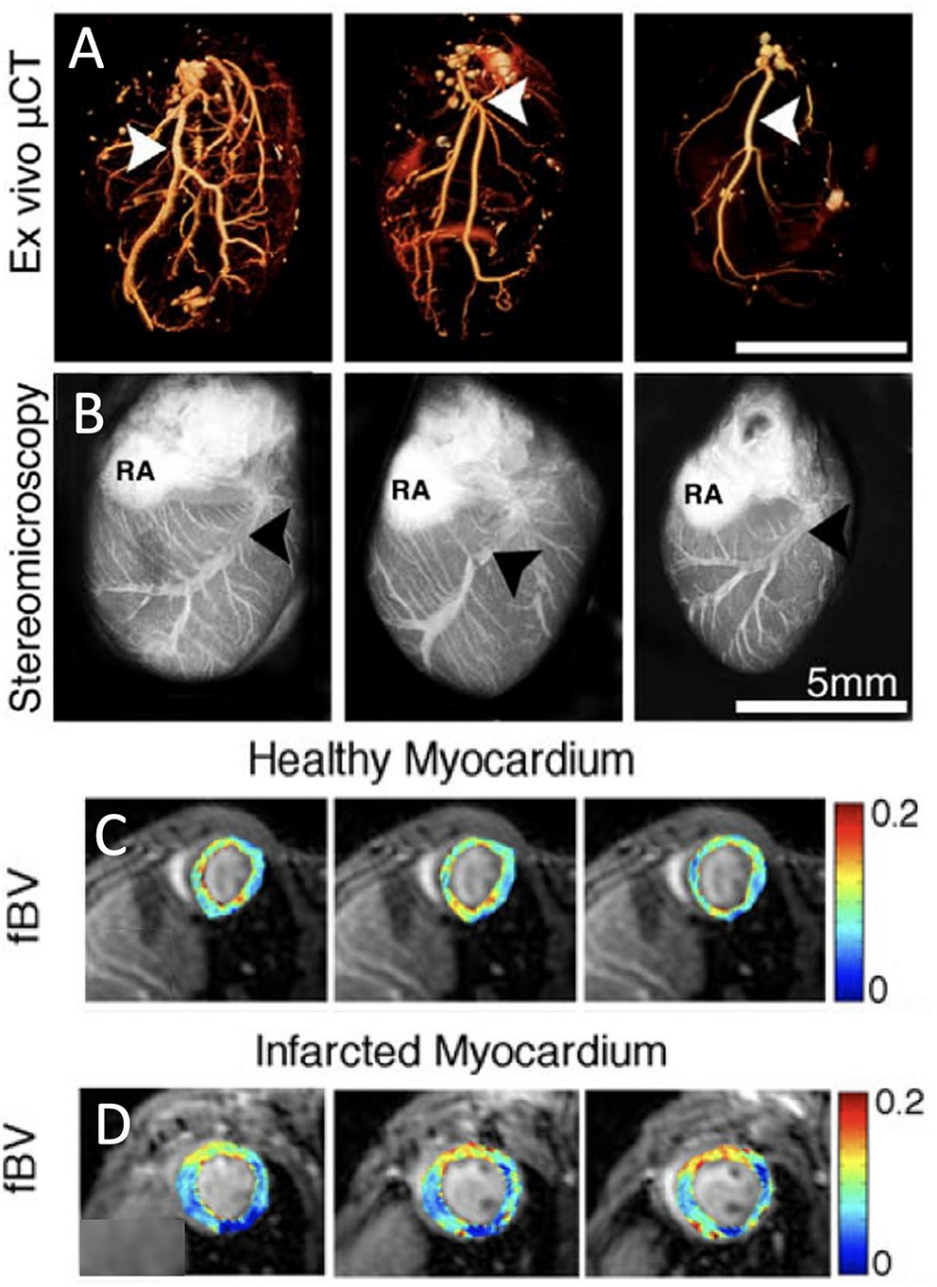
Vessel density and perfusion imaging. **(A)** Volume-rendered *ex vivo* micro-computed tomography (μCT) images of cardiac macrovasculature filled with radio-opaque silicone rubber highlight the left coronary artery (white arrowheads). **(B)** Stereomicroscopic *ex vivo* images of cardiac vasculature after intravenous injection of red-fluorescence-labeled albumin [LCV, left coronary vein (black arrowheads); RA, right atrium; adopted from (21)]. **(C)** Parametric three-dimensional pixel-based maps of fractional blood volume as a measure of microvascular density in healthy myocardium using dynamic contrast enhanced (DCE)-MRI. **(D)** Similarly, DCE-MRI parametric maps of infarcted myocardium [adopted from (22)].

Coronary vessel density can be quantified *ex vivo* by injection of fluorescent blood pool agents for optical imaging (Figures 2A,B) (22) or injection of radio-opaque silicone rubber for micro-CT imaging (Figures 2A,B) (21). This could be of particular interest when studying microvascular vessel density in the heart in mouse models. However, these *ex vivo* techniques do not offer any real-time *in vivo* information and cannot be translated to the clinic.

Lee and colleagues introduced a two-photon method for IVM visualization of blood vessels at subcellular resolution, in the normal murine heart (11) and successfully demonstrated real-time *in vivo* imaging of cardiac tissue dynamics under normal conditions. The technique was also applied in a rat ischemia-reperfusion model, and extracted dynamic biological information on vessel density and vessel structure (23).

Myocardial perfusion imaging is a non-invasive method for the evaluation of blood flow through coronary vessels continuously feeding the pumping heart. After blockage of a coronary vessel, the downstream blood supply in the heart is jeopardized. Standard imaging techniques are used for clinically examining vascular perfusion across the entire heart and assessing the extent of damage to the cardiac muscle. PET and MRI have been widely implemented to monitor blood flow and oxygen supply in absolute blood flow quantification. The absolute quantification of perfusion systematically improves prognostics and diagnostic accuracy.

Myocardial perfusion imaging is also widely used for the assessment and quantification of infarct size in heart disease (20, 24). An *in vivo* mouse study using a clinical SPECT system showed perfect estimation of perfusion defects and non-invasive quantitation of myocardial infarct size (25). Perfusion imaging using either gated blood pool SPECT with ^99m^Tc-sestamibi or SPECT with labeled erythrocytes using ^99m^Tc-pertechnetate has low resolution and is mainly used to non-invasively quantify myocardial infarct size (26). Previous studies have used ^13^N-ammonia for detection of myocardial perfusion, as well as radiolabeled fluorodeoxyglucose (FDG), a glucose tracer, for detection of myocardial glucose metabolism, viability and left ventricular function (26). These methods are also widely used to quantify myocardial perfusion defects in MI patients (27).

In contrast to perfusion SPECT and PET, several MRI techniques have sufficient spatial resolution to quantify the density of blood vessels at the murine myocardium *in vivo*. In both first-pass bolus and macromolecular dynamic contrast enhanced (DCE) MRI, intravenous injection of an MRI contrast agent, followed by MR monitoring of its dynamics through the vasculature of the heart is used to estimate myocardial perfusion. First-pass perfusion MRI using low-molecular-weight Gd diethylenetriaminepentaacetic acid (Gd-DTPA) can identify perfusion delays in the infarcted heart in mice (28) and rats (29). It can also detect myocardial morphology and function, assess viability of cardiomyocytes and predict future myocardial impairment (29). Bolus tracking with Gd-DTPA is widely used for human cardiac perfusion quantification and infarct size measurements (20). To estimate rodent myocardial fractional blood volume, Gd-DTPA-conjugated albumin has been injected and followed with T_1_ mapping. The initial enhancement of DCE MRI at the first time point after injection of macromolecular contrast agent, provides a measure of vessel density, namely the fractional blood volume. In healthy myocardium in C57BL/6 mice cardiac blood volume fraction, evaluating the vessel density, is estimated at around 12%. Three days after MI, a capillary density reduction of ~5% was observed in the infarcted region in a permanent occluded MI model (Figures 2A–D) (22). This perfusion deficit at the infarcted region, was similar to the areas of perfusion deficit shown using other cardiac MRI techniques such as first-pass perfusion (28) and arterial spin labeling (30). Although arterial spin labeling also measures myocardial microcirculatory perfusion, it employs arterial water as an endogenous tracer instead of an injectable contrast agent (11, 22). Chemical exchange saturation transfer (CEST) imaging can also visualize myocardial perfusion following intravenous injection of a contrast agent (31). Mn-enhanced MRI, that has been used in the heart mainly to early detect of abnormalities in myocardial calcium handling, can also quantify viable myocardium pointing out areas with microvascular obstruction. It should be noted though that Mn has toxic effects and has therefore limited translational potential (32). Finally, in patients, contrast-enhanced ultrasound imaging has been applied to quantify microvascular blood flow and blood volume (23). It has been shown effective in assessing blood flow, volume, and flow velocity and to bear translational potential for the safe assessment of microvascular blood flow in the clinic (16).

Although, only single-marker imaging studies of vascular density and perfusion imaging in small animals were presented here, we anticipate vessel density and perfusion imaging to soon be performed using multimodal imaging methods, such as PET/MRI, for a better understanding of CVDs and for clinical translation.

### Endothelial Dysfunction

When stimulated by physiological or pharmacological stimuli, the normal endothelium responds by vasodilating. Early in the pathogenesis of CVD, the production of nitric oxide (NO) by endothelial cells and subsequent vasodilation are compromised (Figure 1C). A malfunctioning endothelium exhibits widespread abnormalities in endothelial integrity, such that permeability changes often coincide with endothelial dysfunction. Endothelial permeability will be discussed in the next section. Although endothelial dysfunction is an early indicator of CVD, it occurs in all stages of vascular disease, such as atherosclerosis, hypertension, diabetes, as well as vascular aging (4). As endothelial dysfunction is an important signature in CVD, the assessment of NO-dependent vasodilation in large and small vessels throughout the body is often used to predict the adverse outcomes of vascular events (5). Identification of impaired endothelium-dependent vasodilation can assist in cardiovascular risk stratification, as it can predict adverse cardiovascular events and unfavorable long-term outcomes.

**Figure 3:**
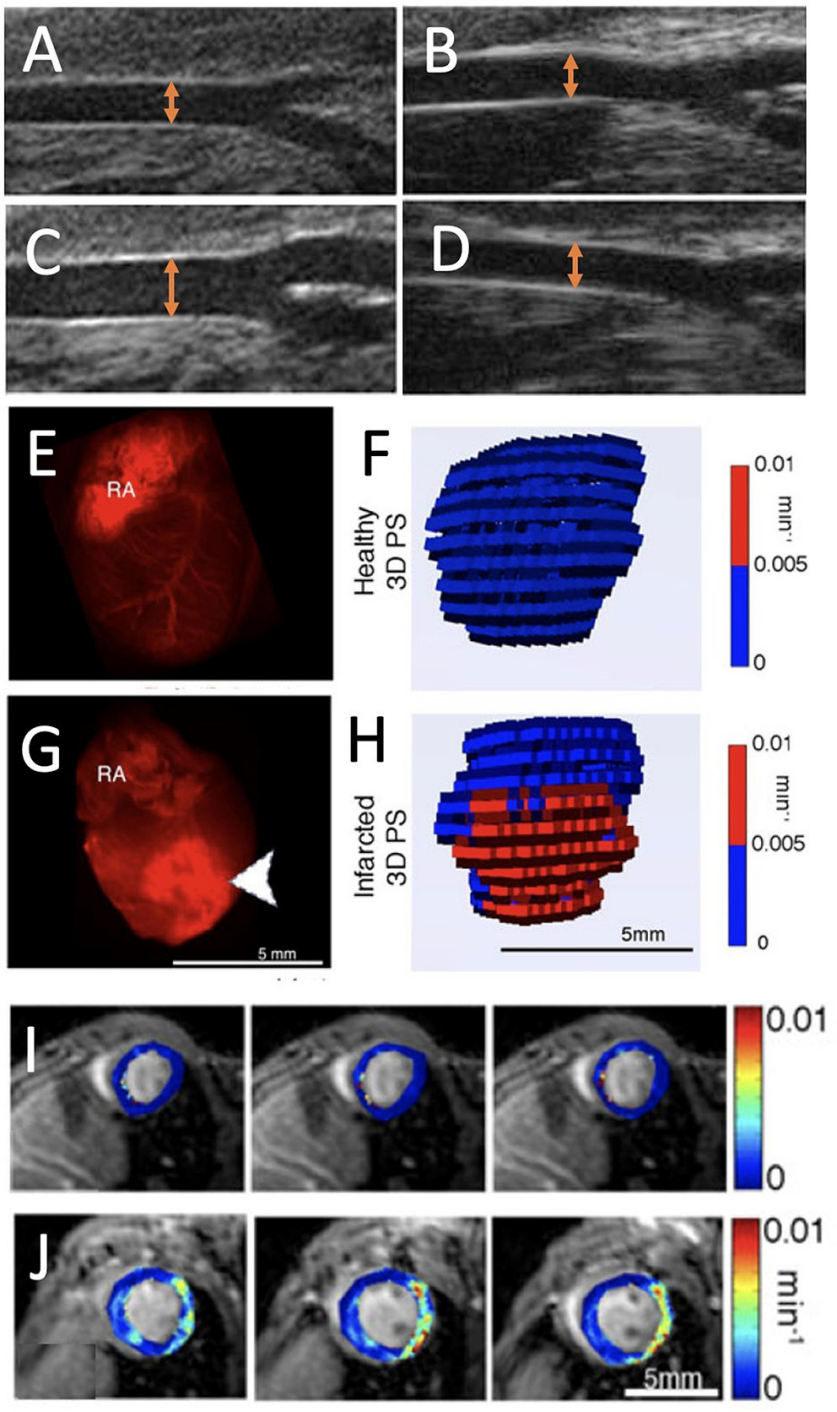
Endothelial dysfunction imaging. **(A,B)** Ultrasound images of *apoE*^−/−^ mouse carotid arteries at baseline and **(C,D)** after a dose of acetylcholine (20 μg/kg/min) on a Western diet [orange arrows represent the artery diameter; adopted from (17)]. **(E)** *Ex vivo* fluorescence images of red fluorescent rhodaminebovine serum albumingadopentetate dimeglumine (rhodamine-BSA-GdDTPA) confined to the blood vessel in healthy myocardium and **(F)** corresponding three-dimensional (3D) visualization of permeability surface area product (PS) in healthy myocardium. **(G)** Extravasated rhodamine-BSA-GdDTPA at the infarct (arrowheads) of an infarcted myocardium (RA, right atrium) with **(H)** 3D representation of PS in infarcted myocardium. **(I,J)** Parametric maps show the absence of permeability in healthy myocardium and PS in infarcted myocardium [adopted from (22)].

Endothelial relaxation has been established by imaging, using ultrasound, PET, and MRI techniques, of vessel dilation following intravenous injection of vasodilators in different mouse models of CVDs. *In vivo* determination of endothelial function in mouse carotid arteries can be achieved by intravenous injection of acetylcholine, followed by ultrasound visualization of vessel relaxation measured by the increased diameter of the arteries. Atherosclerotic mice deficient in apolipoprotein E (*apoE*^−/−^) on a western diet exhibited impaired relaxation *in vivo*. These results matched gold-standard measurements in isolated carotid arterial rings (Figures 3A–D) (17). ^11^C-acetate micro-PET imaging performed in mice exposed to hypercapnia stress was applied to evaluate systemic coronary vasodilatation and endothelial function in the heart and appeared instrumental to assess NO-mediated endothelial response (33). Murine MRI has been demonstrated a sensitive and reproducible tool for detection of artery dilation in response to vasodilators (like acetylcholine) or in response to increased flow. Impaired acetylcholine-induced vasodilation was observed at high-resolution by *in vivo* MRI (34–36). Measurement of endothelial dysfunction in patients by acquisition of pulse-wave Doppler velocity or quantitative CT angiography is well-established (37).

### Vascular Permeability

Cell junctions between endothelial cells are strictly regulated under physiological conditions. While healthy coronary arteries and large vessels have very limited leakiness, in CVDs, tight junctions can loosen in specific regions of the vasculature. Permeability of vessels in the heart and large vessels is triggered by inflammatory or angiogenic cytokines in CVDs, and is associated with the very first stages of angiogenesis but is also accompanied by inflammatory processes (2, 3). Permeable vessels facilitate the extravasation of circulating proteins or molecules toward tissue (Figure 1D). Elevated vascular permeability in CVDs as observed, for example, in infarct healing or atherosclerotic plaques, results in the extravasation of trackable macromolecular molecules (38). Vessel permeability can be imaged using several *in vivo* methods and can be exploited to estimate the cross-endothelial transport of molecules either between endothelial cells (intercellular pathway) and/or through the endothelial cells (transcytosis).

Molecular imaging of endothelial permeability is performed by the assessment of blood pool agents that are generally confined to the intravascular space. Typically, small molecules, such as low-molecular-weight MR-based Gd-chelates, can swiftly extravasate from injured vessels after MI or in atherosclerotic lesions. Delayed-enhancement imaging with MRI consists of the acquisition of one pre-image and one post-image acquired before and after contrast agent injection, respectively. Very rapid diffusion could hamper the evaluation of delicate differences in permeability. However, high-molecular-weight contrast agents, such as labeled albumin, improves the sensitivity of detection of mild elevations in vascular permeability. Loosened tight junctions with intercellular pore size of about 20 nm diameter allow the influx of albumin, an ellipsoid-shaped molecule, with a diameter of 3.8 nm and long axis of 15 nm, into the tissue (39). A time-series of scans is acquired after intravenous injection of either macromolecular agents or agents that bind proteins in the blood.

Epifluorescence whole-heart images, captured 30 min after intravenous injection of labeled albumin, showed albumin's confinement to the blood vessel in healthy myocardium and its influx to the infarcted region (Figures 3E–H) (22). Similarly, a permeable endothelium can be imaged *in situ*, after intravenous injection of Evans blue dye (EBD), which binds to serum albumin in the blood and extravasates in a similar way in atherosclerotic lesions (39). For PET imaging, serum albumin can be targeted *in vivo* with isotopically labeled EBD with ^18^F and ^64^C. This labeling technique has been employed to non-invasively visualize and quantify vascular leakage and permeability in a mouse model of MI (40).

For MRI, the above-mentioned first-pass imaging post-MI can be used to determine perfusion. At a later time point, in a process called late Gd enhancement, which typically occurs 15–30 min after contrast injection, MRI mapping of Gd-DTPA can also define the infarct size (41). This is mainly considered a measure of cardiac injury but is based on the vascular permeability at the infarcted region of the heart. Non-invasive, three-dimensional MRI has been set up to visualize permeability using high-molecular contrast agent in the murine healthy and infarcted heart (22). While low-molecular Gd-DTPA extravasates in any permeable vessel, high-molecular contrast agent can sensitively quantify all shades of permeability from low till high. After MRI quantification of endogenous myocardial T_1_ values, a time series of myocardial scans was performed after intravenous injection of labeled albumin. The vascular permeability in myocardial blood vessels was assessed across the entire heart from the dynamics of injected albumin, and showed increased permeability in infarcted areas. Through the quantification of endogenous myocardial T_1_, the leakage of albumin-conjugated contrast agents, extravasating through permeable myocardial blood vessels, was monitored on short axis cardiac MR images. These permeability measurements identified high vascular permeability in the infarcted region compared to remote regions in the heart 3 days post-infarct (Figures 3I,J). The MRI results were validated using *ex vivo* fluorescence imaging (22). This method has been applied to better understand the effect of statins during early and late phases of infarct healing after MI. Early increases in vascular permeability were observed in impaired atherosclerotic infarct healing. The findings in this study revealed that statins lowered permeability and reduced the transit of unfavorable inflammatory leukocytes into the infarcted tissue, consequently improving left ventricular function (42). Similarly, in a recent work, the efficacy of a regenerative hydrogel loaded with insulin growth factor and vascular endothelial growth factor (VEGF) to ameliorate infarct healing, were assessed by albumin-based DCE-MRI. Altered vascular permeability was observed in the treated infarct regions (43). Lipid-based macromolecular contrast agents, such as liposomes containing Gd-DOTA-lipids, have also been applied in mouse infarct models (44). Because of their similar size, such lipid-based agents are comparable to those of albumin-Gd-DTPA and generated contrast enhancement of the remote myocardium 1 day after an acute MI, as the infarct core remained isointense. T_1_ mapping performed to quantify liposome accumulation in the infarcted area, found that the largest post- and pre-contrast longitudinal relaxation rate difference (ΔR_1_) was in the infarct region (0.60 ± 0.13 s^−1^). Further, in remote tissue (0.15 ± 0.08 s^−1^) and in healthy cardiac tissue (0.23 ± 0.05 s^−1^), the contrast agent exhibited a similar ΔR_1_ (44).

In murine atherosclerotic lesion models, *in vivo* MRI using low-molecular weight Gd, or albumin-binding contrast agents (e.g., Gadofosveset) have been similarly used to detect vascular permeability. In atherosclerotic mice, increased Gadofosveset uptake was observed during plaque progression. Time-dependent changes in relaxation rate of the vessel wall and blood showed high uptake of contrast in the plaque region. Delayed enhancement imaging after Gd-DTPA injection was performed in mice to measure endothelial permeability in the aortic root and to verify reduction in permeability after administration of statin-loaded lipoprotein nanoparticle (45). In parallel, widening of cell-cell junctions and morphological changes of endothelial cells in the region of increased vascular permeability was shown by electron microscopy sections of the plaques (39). Both DCE-MRI and CT imaging are advanced applications for the assessment of vascular perfusion and permeability. DCE-MRI enables two-step pharmacokinetics analysis, comprising intravascular and extravascular component imaging. Initial uptake and first pass of contrast agent in the circulation provide intravascular imaging for perfusion assessment. Subsequent passage of contrast agents to the extravascular space enables delayed imaging and measure of vascular permeability (46). A combined *in vivo* DCE-MRI/CT imaging approach was found beneficial and highly accurate for the assessment of permeability in an acute ischemic stroke study in rats (47). This type of multimodal assessment has not been applied for cardiac or large-vessel imaging, but we believe that, in the future, these types of imaging combinations will provide new avenues for cardiovascular imaging as well.

### Expression of Adhesion Molecules

Adhesion molecules are groups of proteins expressed on the endothelial cell surface that are involved in the multi-step adhesion cascade of leukocytes in CVDs. This adhesion includes initial attachment, rolling, arrest and transmigration of leukocytes from the blood to the underlying tissue. Leukocyte rolling is initiated by the interplay between leukocyte carbohydrate-based ligands, endothelial leukocyte adhesion molecule-1 (E-selectin), and endothelial platelets adhesion molecule-1 (P-selectin). Firm adhesion is driven by the interplay between leukocyte integrin, endothelial intercellular adhesion molecule (ICAM)-1 and vascular cell adhesion molecule (VCAM)-1. Expression of these adhesion molecules and their early detection is important in inflammation and cardiac healing as they play an essential role in tissue repair and immune-vasculature interactions in CVDs. Adhesion molecules can also be involved in thrombus formation (6). Adhesion molecules, like E- and P-selectin or VCAM-1 and ICAM-1, mark early signs of an activated endothelium and can be specifically targeted and imaged in CVDs (Figure 1E). *In vivo* optical, ultrasound, PET and MRI imaging techniques have been employed to assess these biomarkers in CVDs (19).

**Figure 4:**
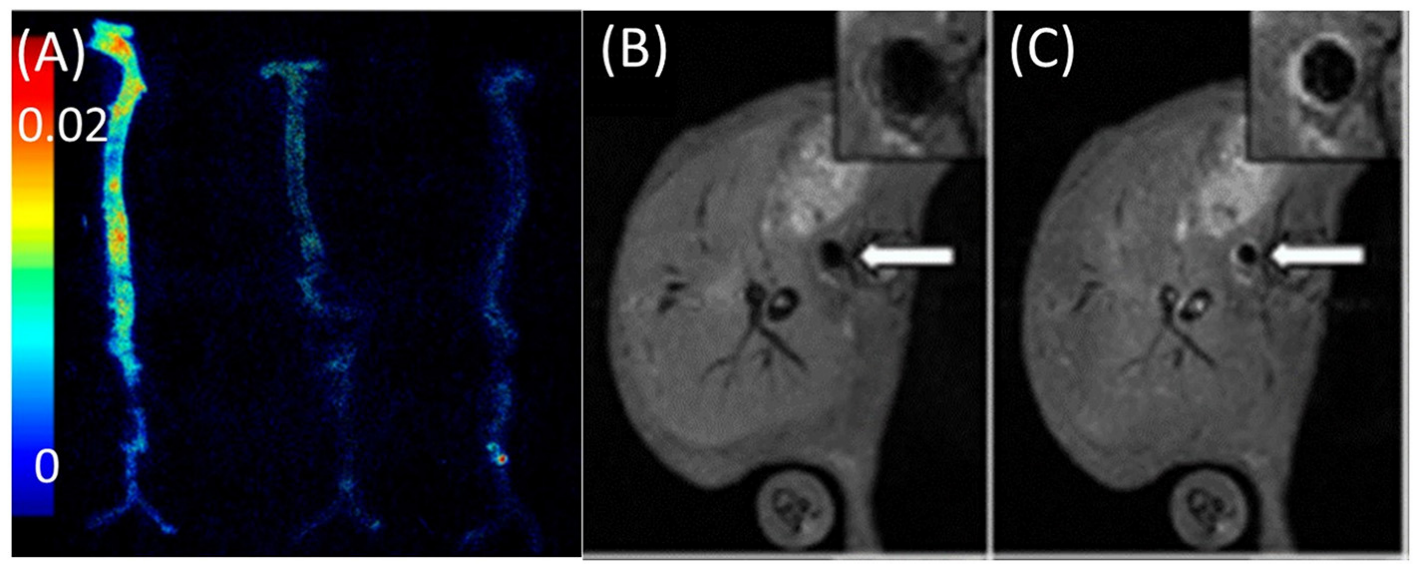
Imaging of adhesion molecules. **(A)** *Ex vivo* fluorescence image of aortas obtained by VCAM imaging of *apoE*^−/−^ mice injected with (from left to right) VCAM-TMV, PEG-TMV, and PBS, respectively. **(B)** Pre-injection and **(C)** post-injection MRI scans of VCAM-TMV in *apoE*^−/−^ mice. Insets show magnified images of abdominal aorta with regions of interest [adopted from (12)]. VCAM, vascular cell adhesion molecule; PEG, Polyethylene glycol; TMV, Tobacco mosaic virus.

Cell-adhesion molecules cooperate in the early advancement of atherosclerotic lesions by assisting in leukocyte recruitment to the vessel wall. A dual optical and MRI imaging approach applied to visualize VCAM-1 targeting using both near-infrared dyes and chelated Gd ions demonstrated its utility in targeting adhesion molecules in a murine atherosclerotic *apoE*−/− model. The team demonstrated delivery of contrast agent to atherosclerotic plaque regions by targeting cell receptors present on endothelial cells. *Ex vivo* fluorescence and *in vivo* MR imaging showed the uptake and targeting ability of the contrast agent in aortas and the abdominal aorta (Figures 4A–C) (12). This imaging method for detection and quantification of VCAM-1 allows for early identification of inflammation in atherosclerosis. Similar real-time detection of VCAM-1 expression was employed to identify activated cells in human plaques. Research aligning with these studies, was performed in *apoE*^−/−^ mice, in which contrast agent uptake in developing plaques was dynamically evaluated for 28 days. High-contrast uptake in the region of VCAM-1 expression by endothelial cells was quantified in atherosclerotic plaques with high affinity and sensitivity. This enabled real-time assessment of VCAM-1 expression in atherosclerosis, which served as a proxy for activated cells in human plaques (48).

Using an ultrasound-based strategy, microbubbles designed to target the adhesion molecule ICAM-1 have been injected and imaged to detect inflammation and consequent signs for transplant rejection of heterotopic hearts in rats (18). Progressive atherosclerotic plaques exhibit active expression of P-selectin during activation of the endothelium overlying active atherosclerotic plaques. Inactive fibrous plaques do not exhibit expression of P-selectin. *In vivo* characterization of P-selectin expression by PET imaging of active plaques was achieved with ^68^Ga-Fucoidan, a highly sensitive polysaccharidic ligand of P-selectin (49). Furthermore, ^64^Cu-labeled anti-P-selectin monoclonal antibodies were used to target endothelial activation at atherosclerotic lesions in low-density lipoprotein receptor-deficient Ldlr^−/−^ mice on a high-cholesterol diet. PET/CT fusion imaging revealed selective and prominent accumulation of the probe in the aortic root. *Ex vivo* autoradiography of aortas additionally confirmed probe uptake (50).

Targeting of E-selectin using an appropriate contrast agent, together with visualization of immune cell recruitment and their interaction with endothelial cells, was performed both *in vitro* and *in vivo* models of hepatitis in mice. The targeted contrast agent, particularly iron nanoparticles, recognized E-selectin and bound extracellularly to E-selectin overexpressed on activated endothelial cells, thus facilitating *in vivo* MRI of endothelial cell activation (51). In a mouse model of inflammation, the distribution of E-selectin expression on an activated vascular endothelium and its localization was validated using iron nanoparticles; histological analysis confirmed their distribution (52). Two MRI studies used iron nanoparticles to target VCAM-1 on activated endothelium in atherosclerotic *apoE*^−/−^ mice (53, 54). The VCAM-1-targeted iron nanoparticles accumulated in plaques, which resulted in negative contrast enhancement, highlighting plaques rich in macrophages. VCAM-1 targeting appeared very sensitive, as a low dose of contrast agent was sufficient to identify activated plaque regions (53). In addition, the accumulation of VCAM-1-targeted iron nanoparticles in atherosclerotic lesions highlighted the applicability of atherosclerosis imaging with MRI (54).

Imaging of adhesion molecules in vascular processes is important because it zooms in to areas in the endothelium that harness the recruitment of immune cells, which is crucial in both cardiac healing and atherosclerotic progression.

### Vessel Wall Inflammation

When adhesion molecules are expressed, leukocyte-like neutrophils, monocytes and macrophages can transmigrate through the endothelial lining and cause vessel wall inflammation. After intravenous injection, nanoparticles are internalized by leukocytes, primarily by monocytes, macrophages, and less so by neutrophils. Once in the tissue, the leukocytes with internalized nanoparticles, can be imaged as they transmigrate into the vessel wall. In this way, the progression of vessel wall inflammation can be non-invasively tracked (Figure 1F).

The uptake of fluorescently labeled iron nanoparticles by macrophages has been imaged in infarcted myocardium using both *in vivo* optical imaging and MRI. The fluorescent signal throughout the heart was quantified *via* fluorescence molecular tomography images of mice with MI. Besides fluorescence imaging, non-invasive infiltration of iron nanoparticles at the infarcted areas of the heart was shown as a hypointense signal using MRI. This could be of significant value in both preclinical and clinical settings. The techniques developed can also be used to image other existing fluorescent and magneto-fluorescent probes and can significantly expand the role of fluorescence imaging in the heart (55). Another study in mice quantified the uptake of iron-nanoparticle-based contrast agents in plaque regions in cardiac arteries using *in vivo* MRI. MRI mapping and accumulation of the contrast agent showed that average T_2_ values in the plaque region decreased from a baseline value of 34.5 ± 0.6 ms to 24.0 ± 0.4 ms 1 day after injection. The T_2_ values were inversely related to the amount of iron nanoparticles in the plaque region assessed by *ex vivo* particle electron paramagnetic resonance. Furthermore, plaque progression and treatment outcomes were assessed and monitored (56). Similarly, visualization of emulsified perfluorocarbon, by *in vivo* fluorine (^19^F) MRI in an acute cardiac ischemia murine model showed its significant uptake in lymph nodes and identified that circulating monocytes/macrophages are the main cell fraction contributing to image contrast. This MRI-based approach allows numerous applications of inflammatory disease states (57).

Iron nanoparticles have also been applied to track macrophage-rich atherosclerotic plaques using positive-contrast MRI in small animals. Iron nanoparticle uptake and signal enhancement were found specifically in inflamed atherosclerotic plaques (58). In hyperlipidemic rabbits, iron nanoparticle-based contrast agents were shown to accumulate in plaques with high macrophage content and generated significant contrast and signal changes in infected regions (59). Macrophage recruitment in acute atherosclerosis was also studied, and quantification of macrophages directly correlated with dynamically monitored vascular changes in plaque inflammation. Moreover, *in vitro* validation results displayed concentration-dependent contrast agent uptake by macrophages (60).

In a recent study, nanoparticles were used for macrophage imaging in inflamed cardiovascular tissues using multi-scale imaging. It was shown that ^18^F-Macroflor, a modified nanoparticle with high avidity for macrophages, was enriched in cardiac and plaque macrophages. This enabled the sensitive tracking of macrophages in a PET/MRI study of the infarcted heart in both mice and rabbits (61). Another multimodal strategy was recently implemented and applied to quantify ^89^Zr-labeled liposomal nanoparticle uptake in the atherosclerotic vessel wall of rabbits using PET, in conjunction with CT and DCE-MRI with low-molecular Gd-DTPA for vascular permeability. The analysis showed that uptake was significantly higher in atherosclerotic compared to control rabbits (1.8 vs. 0.9 g/mL). Nanoparticle biodistribution and vessel wall targeting in a rabbit atherosclerosis model was also evaluated *ex vivo* using optical imaging and autoradiography. The study provided a multimodal tool to accurately validate nanoparticle targeting of plaques in atherosclerotic vessel walls (62).

Dextranated and DTPA-modified magneto-fluorescent nanoparticles labeled with PET tracer ^64^Cu were used for *in vivo* multimodal imaging to directly detect macrophages in atherosclerotic plaques of *apoE*^−/−^ mice. This new PET-based contrast agent with avidity for macrophages was developed with optimized pharmacokinetics to allow for *in vivo* imaging of macrophage recruitment to injured aortas. The contrast agent facilitated the quantification of an elevated number of macrophages in *apoE*^−/−^aortas compared to wild type aortas. The study provided an improved imaging probe with enhanced sensitivity and direct correlation to PET signal intensities and localization (63). Of note, intravenous injection of iron nanoparticles may change the course of infarct healing after MI, as these nanoparticles have proven cardioprotective features (64).

### Thrombosis

During homeostasis, endothelial cells are key regulators of thrombosis as they produce anticoagulant and antithrombotic factors, such as prostacyclin, NO and plasminogen activator and its inhibitor. After activation or injury of endothelial cells, they become procoagulant and prothrombotic (10). Thrombosis is the formation of blood clots, which are comprised of platelets and fibrin, inside a vessel. In this process, endothelial cells release von Willebrand Factor, a blood glycoprotein involved in hemostasis that assists with platelet adhesion to exposed matrix, and eventually to blood flow obstruction. Atherosclerotic plaques that exhibit thrombo-modulatory factors have been shown to be less stable (10), thus making thrombosis a key process to that can serve as a marker of unstable plaques (Figure 1G).

The process of cellular thrombosis formation has been assessed using fluorescent probes *in vivo* as well as in an *ex vivo* flow chamber system. Fluorescein isothiocyanate (FITC)-labeled isolectin at the endothelial cells, whereas circulating platelets were fluorescently stained by rhodamine 6G. After *ex vivo* isolation of cremasteric arteries of mice, thrombus formation after functional damage to the endothelium was visualized (65). Complement C3 is a factor that is involved in thrombosis and has been targeted for imaging; non-invasive ultrasound imaging displayed serum complement C3 adherence of albumin-encapsulated microbubbles to the vascular endothelium at atherosclerotic plaques in *apoE*^−/−^ mice. Microbubbles were also fluorescently labeled so that the adherence to activated endothelium of atherosclerotic plaque could be confirmed by fluorescent microscopy (66).

**Figure 5:**
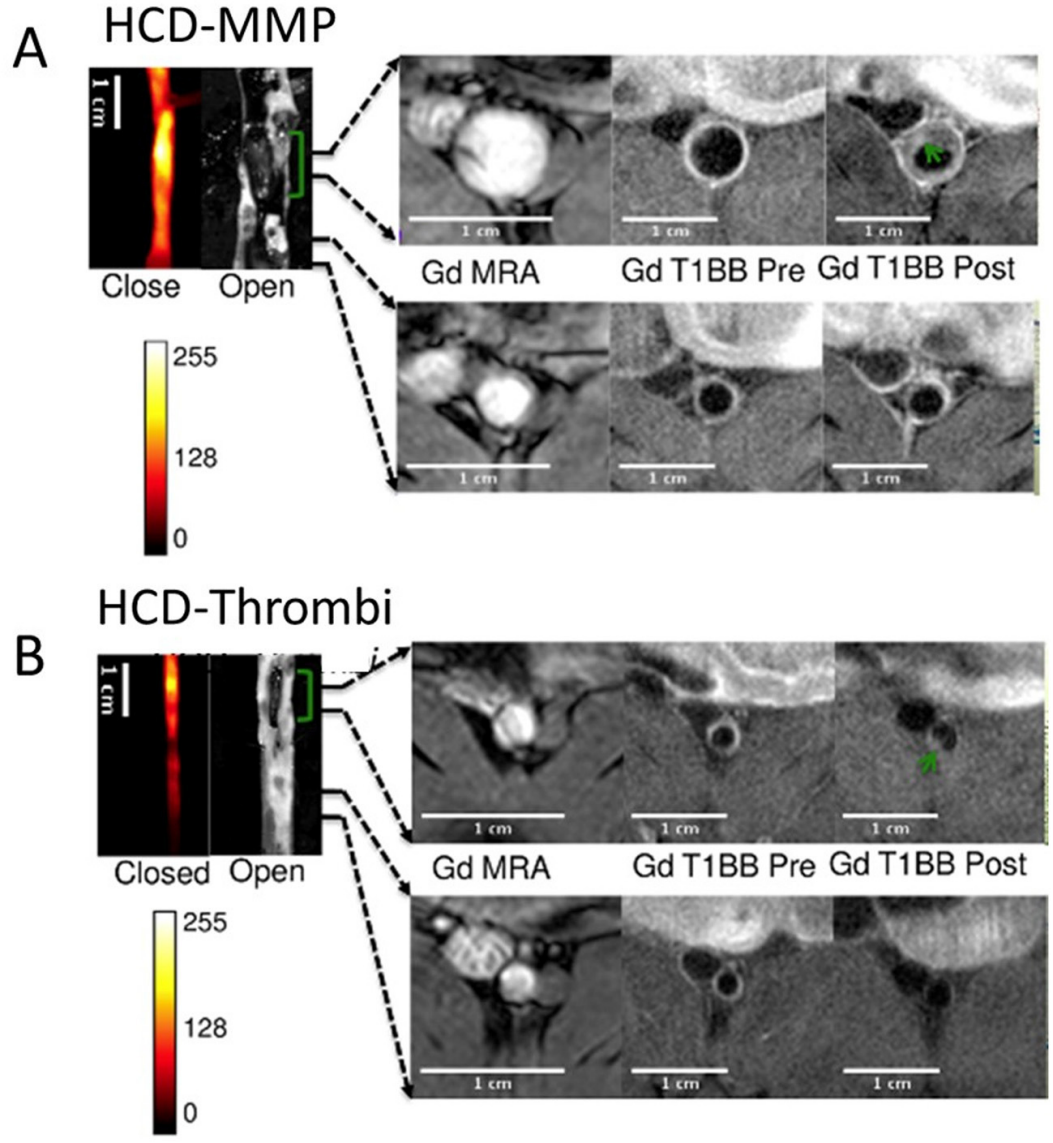
Thrombus imaging. Fluorescence and MRI images of rabbits collected using a **(A)** HCD+MMP-ACPP probe and **(B)** HCD+ Thrombin-ACPP probe, respectively. The closed view of the fluorescence images shows a sub-section of the abdominal aorta and the corresponding open view reflects images showing the location of thrombi (green brackets). The regions within the two black lines represent examples of non-disrupted and disrupted plaques, respectively, and correspond to the MRI images on the right. All MRI images were obtained after injection of Gd-DTPA according to acquisition protocols. Illustrated are the pre-triggering magnetic resonance angiography (MRA) and the fat suppressed T_1_BB images obtained before and after pharmacological triggering (from left to right). Thrombi are marked by green arrows in post-triggering Gd-T_1_BB (T_1_ black blood) images [adopted from (75)]. HCD, high-cholesterol diet; MMPs, matrix metalloproteinases; ACPP, activatable cell-penetrating peptides; Gd, DTPA, gadolinium chelate.

In atherosclerotic rabbits, plaque rupture with subsequent thrombosis was imaged with MRI using fibrin-binding Gd-labeled peptide (EP-1873) (67). A fibrin nanoagent has been targeted and labeled for both MRI and optical imaging using iron nanoparticle and near-infrared fluorescence agent, respectively, to assess and detect microthrombosis in a rat MI model (68). Fibrin is a major constituent of arterial and venous thrombi and has been targeted in several multimodal imaging methods of thrombosis (69–71). A recent ^19^F MRI study showed the selective targeting of activated platelets using activated integrin glycoprotein IIb/IIIa labeled with perfluorocarbon, in both mice and humans (72). The fibrin-binding probe FBP7 labeled with ^64^Cu has been developed for PET, to detect both arterial and venous thrombi in rats with either carotid crush injury (mural thrombosis model) or embolic stroke (occlusive thrombosis model) (69). Multisite thrombus imaging and estimation of fibrin content were also achieved with whole-body PET in similar studies (69, 70, 73). Furthermore, *in vivo* multimodal imaging was performed using MRI/PET/SPECT techniques to detect and visualize vascular injury and thrombogenic plaque in mice using ^64^Cu-labeled glycoprotein VI-Fc ^64^Cu-GPVI-Fc contrast agent. *Ex vivo* histology and *in vivo* MRI and PET imaging results were validated and collagen content, plaque burden and vessel wall functioning in the endothelial cell injury and thrombus detection were measured. Uptake of ^64^Cu-GPVI-Fc was found higher in the injured left carotid artery wall than in the intact right carotid artery. As validated in *apoE*^−/−^ mice, ^64^Cu-GPVI-Fc uptake in the aortic arch was significantly higher compared to wild type mice (*apoE*^−/−^: 13.2 ± 1.5 Bq/cm^3^ vs. wild type mice: 5.1 ± 0.5 Bq/cm^3^, *P* = 0.028). Similarly, high relaxation rates were measured in the injured carotid wall in T_1_ MR scanning (injured carotid wall: 1.44 ± 0.08 s^−1^ vs. intact carotid wall: 0.91 ± 0.02 s^−1^; *P* = 0.028) (74). In a more recent study, fluorescently labeled, activatable, cell-penetrating peptides (ACPPs) were designed and used to visualize there *in vivo* uptake, after their cleavage by matrix metalloproteinases (MMPs) or thrombin. ACPP uptake, mapped *ex vivo* in the whole aortas, was higher in disrupted compared to non-disrupted plaques. The combination of optical molecular and Gd-enhanced MRI imaging for detection and assessment of endothelial defects, facilitated the identification of high-risk unstable plaques in atherothrombosis (Figures 5A,B) (75).

### Angiogenesis

Angiogenesis is a complex molecular and cellular process that involves the proliferation of endothelial cells after cardiac ischemia and during atherosclerotic plaque development. The process of angiogenesis entails not only endothelial cell activation by pro-angiogenic factors, but also increased permeability, as discussed above, and ECM remodeling (3, 7). Although ECM remodeling is key to the development of new vessels, it will be discussed separately below, as the ECM can also remodel in fibrotic processes with little connection to angiogenic changes. Targets for pro-angiogenic imaging in infarct healing, and in atherosclerotic plaques include VEGF, its receptor (VEGFR) and α_*v*_β_3_ integrin on activated endothelial cells. In response to an ischemic lesion, VEGF is released at the myocardium or in plaques to stimulate the formation of vessels to supply oxygen and nutrients (Figure 1H). Activation of the α_*v*_β_3_ integrins on the ECM side of the endothelial cells, is essential for endothelial cell propagation. Short peptide sequences, like Arg-Gly-Asp (RGD), can recognize and label α_*v*_β_3_ integrin activation for several imaging modalities. Although the main imaging markers for angiogenesis are VEGF and α_*v*_β_3_ integrin, we will also point out some other pro-angiogenic imaging strategies (76, 77).

#### Angiogenesis in Infarct Healing

After MI, a large area of hypoxic tissue within the heart initiates the expression of various pro-angiogenic genes in endothelial cells. Angiogenesis is an important biological process that occurs during the proliferative phase of myocardial healing after the infarct. Tracking the angiogenic responses is crucial when monitoring the response of healing after infarction, particularly in the assessment of efficacy and safety of novel regenerative treatments after MI. Recent progress in molecular imaging has brought to the development of a platform to visualize the formation of new blood vessels using optical, ultrasound, SPECT, PET, and MRI imaging approaches *in vivo* (78).

Angiogenesis is an integral part of scar formation after MI. Apln is expressed in response to hypoxia and has been employed to visualize sprouting blood vessels at the MI border zone using confocal microscopy (79). In addition, after an ischemic event, the pro-angiogenic factor VEGF and its receptor VEGFR are highly expressed and serve as targets for imaging myocardial angiogenesis. In stroke, bioluminescence imaging with luciferase has been used to image VEGF receptor 2 (VEGFR2). A novel approach for optical quantification of angiogenic sprouts after MI involves study of the apelin (Apln) reporter mice (79). Transgenic mouse expressing firefly luciferase under the control of the VEGFR2 promotor were utilized to non-invasively decipher the temporal profile of VEGFR2 expression after stroke highlighting VEGF/VEGFR2 signaling (13).

Furthermore, *in vivo* ultrasonography imaging of VEGFR2 expression on endothelial cells was used for molecular and functional assessment in a mouse hind limb ischemia model (80). In murine hind-limb ischemia study, ^64^Cu-VEGF_121_ has been employed to image angiogenesis by PET. In this study, VEGF was upregulated following treadmill exercise training (81). In a rat model of MI, the same ^64^Cu-VEGF_121_ tracer was tracked by *in vivo* cardiac PET imaging. This dynamic study showed that VEGF levels peaked 3 days after MI and subsequently declined over time, reaching baseline levels 24 days after MI (82). For tumor imaging, both VEGFR-1 and VEGFR-2 receptors were exploited as PET radiotracers and successfully validated the mapping of angiogenesis (83). In the future, we envision that these types of studies, can also be applied in cardiac vascular imaging after MI to reveal dynamic biological processes.

**Figure 6:**
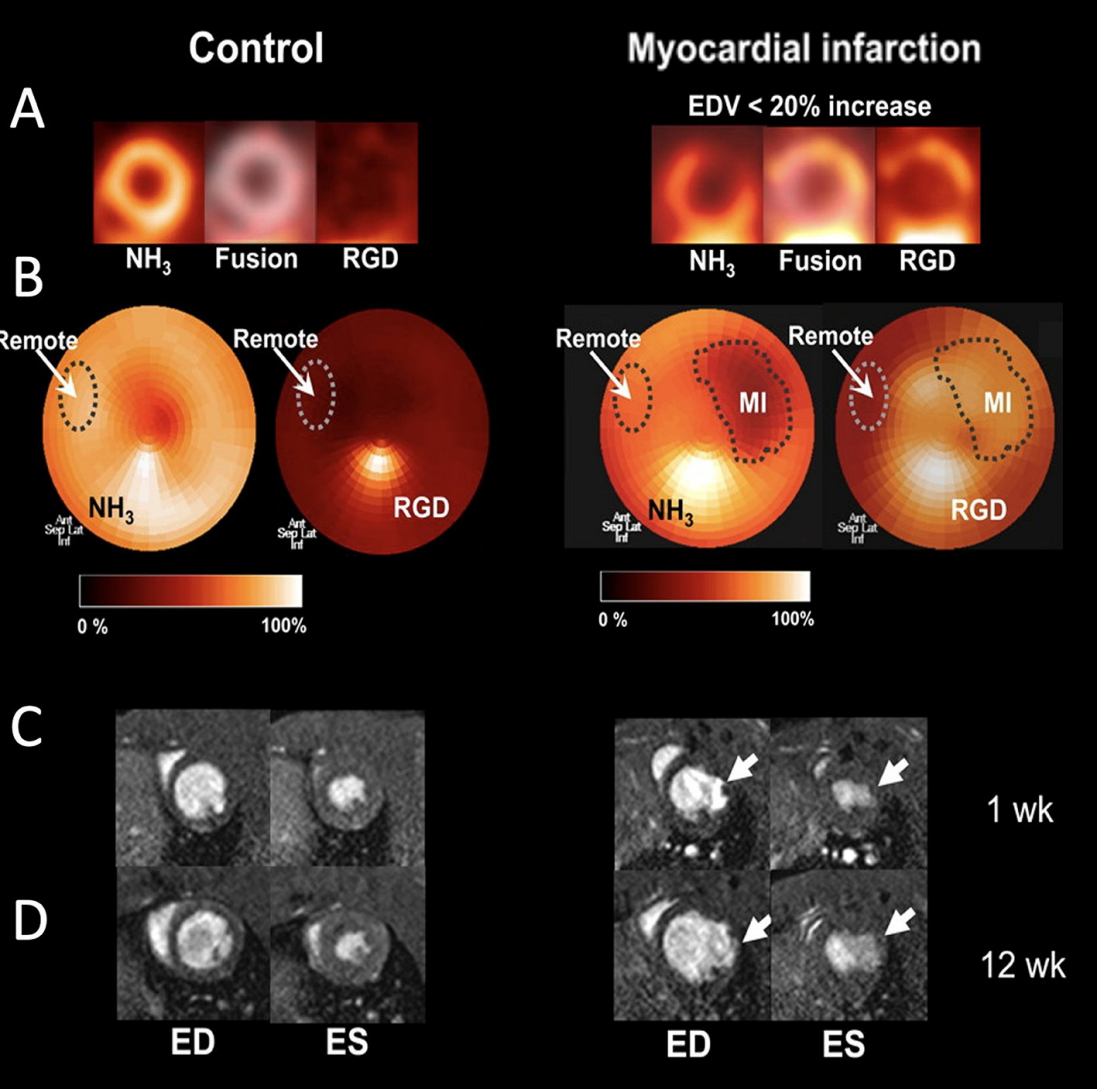
Imaging of angiogenesis in myocardial infarction (MI). **(A)** PET images of control rats and rats after MI (right), which show separate and fused co-registered NH_3_ and ^18^F-RGD tomograms. **(B)** NH_3_ polar maps and co-registered ^18^F-RGD polar maps. Each NH_3_ polar map was normalized to its corresponding remote myocardium uptake and defect area region of interest (ROI) was defined using 50% threshold. Subsequently, remote and defect ROIs were co-registered in the ^18^F-RGD polar map using same axes to evaluate ^18^F-RGD uptake in infarcted and remote myocardium. **(C)** Representative cross-sectional MR images of the left ventricle (LV) during end-diastole (ED) and end-systole (ES) 1 week after MI. **(D)** Corresponding MR images of same rats 12 week after MI [adopted from (88)]. ^18^F-RGD, ^18^F-galacto-RGD.

Another approach used to image angiogenic processes targets the transmembrane protein α_*v*_β_3_ integrin that can be imaged with labeled RGD after acute MI (77). For instance, a targeted radiotracer of α_*v*_β_3_ integrin was utilized to dynamically track angiogenesis in a murine limb ischemia model. More specifically, a ^99^Tc-labeled peptide that binds to α_*v*_β_3_ integrin, was employed to follow the activation of α_*v*_β_3_ integrin over time at the ischemic region (84). In the same hind-limb model, a multimodal approach was used to assess angiogenesis by labeling the α_*v*_β_3_ integrin-binding peptide not only for SPECT imaging (by ^99^Tc), but also for *in vivo* fluorescence imaging (85). Serial *in vivo* SPECT imaging has been performed in both rat and canine MI models using a ^111^In-labeled α_*v*_β_3_-targeting agent. This approach demonstrated increased myocardial radiotracer uptake in the infarcted region, indicating myocardial angiogenesis (86). Its uptake in the infarcted myocardium coincided with regions of hypoxia, as imaged *in vivo* due to elevated levels of the hypoxic marker ^111^In-RP4748 (87). Additionally, ^18^F-RGD has been applied in a rat model of MI. One week after permanent left coronary artery (LCA) ligation, rats were injected with ^18^F-RGD to evaluate α_v_β_3_ integrin expression in the infarcted area using a small-animal PET scanner. In the same rats, changes in LV cavity size, LV function, and infarct size were studied by serial ^13^N-ammonia PET and MRI measurements at 1 and 12 weeks after MI. Uptake of ^18^F-RGD was compared with the presence of angiogenesis in histologic samples at 1 week after MI in a subgroup of rats. Increased uptake of ^18^F-RGD in the perfusion defect area early after MI correlated with improved cardiac function and eventual outcome (Figure 6) (88). These imaging studies have marked α_v_β_3_ integrin as a biomarker of myocardial repair processes after MI. This combination of PET/CT molecular imaging with functional cardiac MRI has found its way to the clinic. Multimodal imaging of cardiac α_V_β_3_ integrin in patients after acute MI has been performed using PET/CT/MRI imaging (83, 84). Finally, multiparametric PET and MRI using α_V_β_3_ integrin as a target have been used in infarct patients to predict long-term cardiac function. Imaging of elevated angiogenic markers in infarct healing has been demonstrated an important prognostic approach after MI (89).

#### Angiogenesis in Atherosclerotic Plaques

Plaque angiogenesis and rapid growth of the vasa vasorum in the vessel wall, have been associated with the development of unstable plaques, particularly in patients with diabetes and unstable coronary syndrome. Neovascularization of the plaque can cause plaque rupture through several mechanisms. Immature vessels increase permeability and protease activation, which, in turn, could lead to hemorrhages from plaque capillaries, induction of inflammation and plaque instability (85, 86). *In vivo* tracking can be exploited to better understand the involvement of angiogenesis in the pathophysiology of atherosclerosis, by quantifying pro-angiogenic markers along large vessels.

The advancement of unstable plaques is associated with increased VEGF/VEGFR signaling and is enhanced in diabetes (90). *Ex vivo* near-infrared fluorescence imaging with anti-VEGF-800CW, identified VEGF-A overexpression in atherosclerotic plaques (91).

A VEGF-based SPECT tracer labeled with ^99^Tc was shown to identify accelerated atherosclerosis in diabetic atherosclerotic *apoE*^−/−^ mice (92). The same *apoE*^−/−^ mice were used to test statin therapy, known to stabilize atherosclerotic plaques. These mice were probed by non-invasive SPECT imaging of atherosclerotic neovascularization using a ^99^Tc-labeled anti-VEGF antibody. This imaging strategy allowed for the non-invasive diagnosis and assessment of plaque neovascularization, showing the antiangiogenic effect of atorvastatin (93).

**Figure 7:**
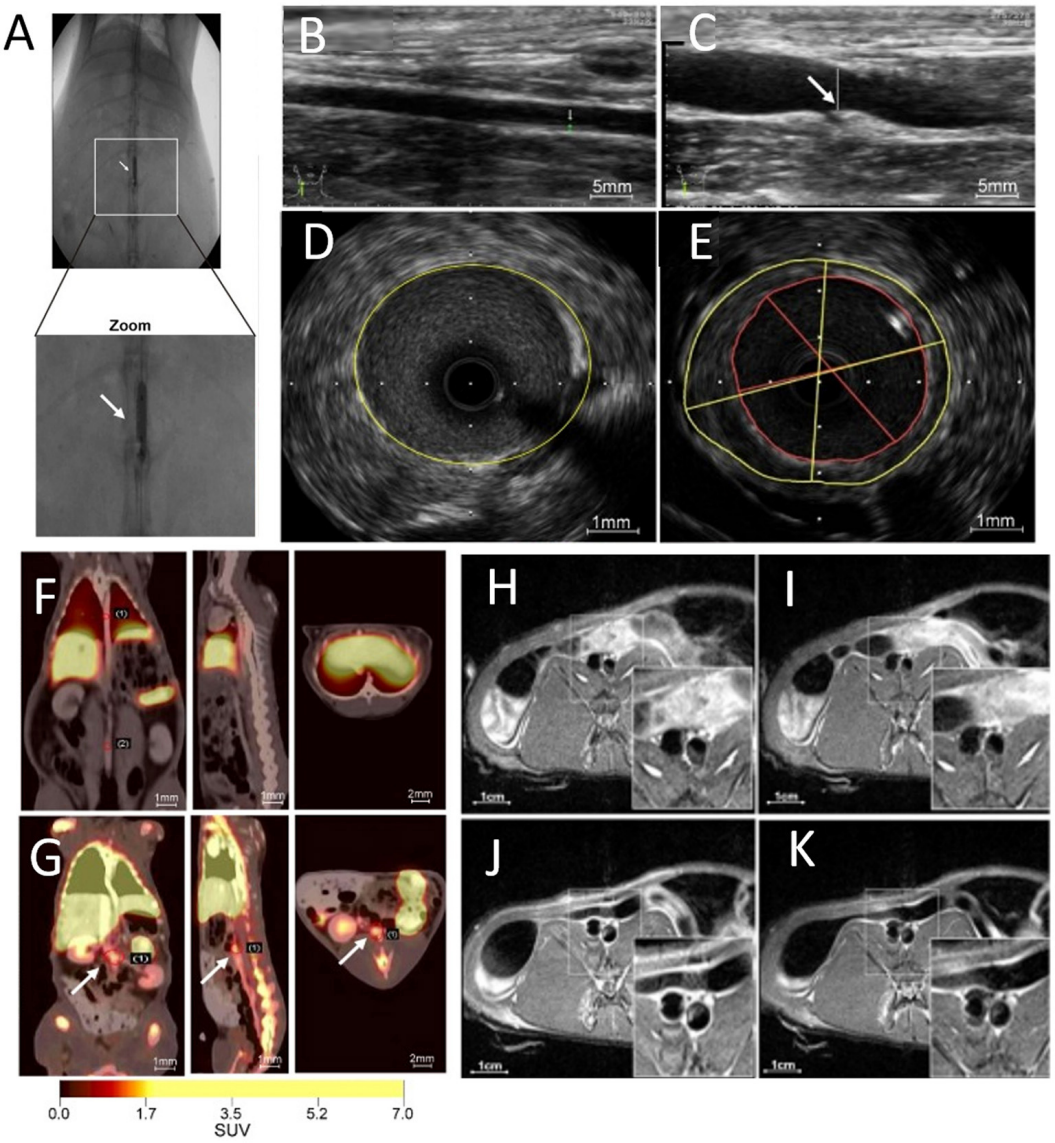
Multimodal imaging of angiogenesis. **(A)** Abdominal aorta balloon denudation model, showing ultrasonic images of **(B)** control vessels and **(C)** vessels after denudation injury displaying vessel enlargement and plaque formation 8 weeks after injury. Representative intravascular ultrasonic images of **(D)** control and **(E)** injured vessels, showing a greater plaque burden and plaque eccentricity in the injured mice. PET images of rabbits 2 h after **(F)** ^68^Ga-NGD-MNPs or **(G)** ^68^Ga-NUD-MNPs injection. MR images of abdominal aorta before and 4 h after **(H,I)** control NUD-MNPs injection or **(J,K)** GEBP11 peptide targeted NGD-MNPs injection [Scale bar (H-K)−1 cm; adopted from (95) with pending permission]. ^68^Ga-NUD-MNPs, ^68^Ga-NOTA-unrelated peptide-DMSA-MNPs; ^68^Ga-NGD-MNPs, ^68^Ga-NOTA-GEBP11-DMSA-MNPs; DMSA-MNPs, 2, 3-dimercaptosuccinnic acid-coated paramagnetic nanoparticles; NOTA, 1,4,7-triazacyclononane-N,N',N”-triacetic acid; GEBP11, peptide with angiogenic affinity.

Plaque angiogenesis has also been non-invasively probed using integrin α_v_β_3_ ligands. Following intravenous injection of α_v_β_3_-integrin–targeted paramagnetic nanoparticles into atherosclerotic rabbits, increased MRI signals were recorded at the regions of the neovasculature, specifically throughout the abdominal aortic wall (94). Finally, atherosclerotic plaque angiogenesis in the abdominal aorta was also probed using PET/MR imaging and ^68^Ga-labeled, GEBP11 peptide-targeted magnetic iron oxide nanoparticles in a rabbit model of atherosclerosis (Figure 7) (95).

### Extracellular Matrix Rearrangement

The ECM is critical for all aspects of vascular biology. Together with supporting cells, endothelial cells form a basement membrane matrix contributing to structural and organizational stability of the vasculature. In the progression of CVDs, the basement membrane matrix supporting the endothelium can be degraded by matrix metalloproteinases (MMPs) (Figure 1I). The ECM supports essential functions of endothelial cells (8). For example, in angiogenesis, the ECM facilitates migration, invasion, proliferation, and survival of endothelial cells, as it serves as a scaffold protecting and guiding the endothelial cells. Subsequently, integrins are activated, as described in the angiogenesis section (8). Proper balance between proteolytic activities and ECM production is pivotal in cardiovascular health (96).

#### Matrix Metalloproteinases

MMPs are a family of zinc-dependent endopeptidases that play an essential role CVDs. By degradation of the surrounding matrix, MMPs facilitate angiogenesis as they clear the way for endothelial cell to grow and migrate. Likewise, they rearrange the ECM in the heart and the vessel wall during CVDs. Therefore, MMPs and their enzymatic activities are important targets for optical, SPECT, PET, and MRI imaging.

**Figure 8:**
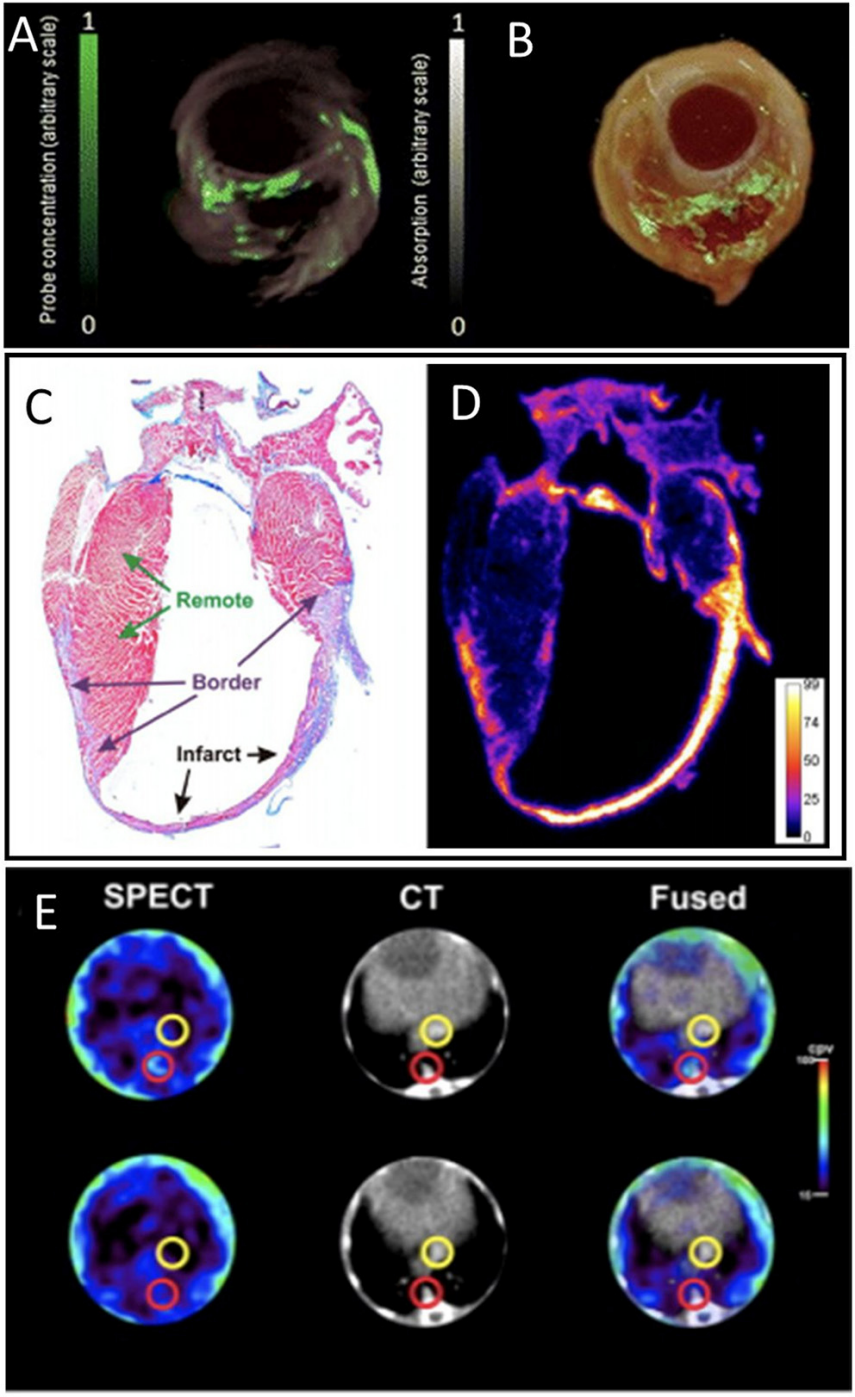
Matrix metalloproteinase (MMP) activity imaging. **(A)** Imaging of intact plaques made using deep-tissue multispectral optoacoustic tomography. Cross-sectional multispectral reconstruction, revealing the location of MMPSense 680 activity in the slice (green) superimposed onto morphological optoacoustic images. **(B)** The corresponding epi-fluorescence images of a dissected plaque (in green) superimposed onto color images of cryosections from the three carotid plaque specimen [adopted from (15)]. Histology and autoradiography of a coronal section of the mouse heart 20 h post-injection of ^177^Lu-ACPP 10 days after myocardial infarction. **(C)** Representative azan staining showing infarct scar in blue and remote myocardium in red. **(D)** Autoradiography shows enhanced uptake of ^177^Lu-ACPP in the infarct zone in an adjacent section [adopted from (98)]. **(E)** Transversal SPECT, CT, and fused SPECT and CT images of MMP activation using RP782 in atherosclerotic *apoE*^−/−^ mice, demonstrating areas of high (top) and low (bottom) tracer uptake in aorta (red circles). Slight tracer uptake can be detected in IVC [yellow circles; adopted from (99)]. ACPP, Activatable cell-penetrating peptides; RP782, ^111^In-labeled tracer specific to MMP activity.

A near-infrared fluorescent probe, activated by proteolytic cleavage by MMP2 and MMP9, has been characterized for optical imaging of MMP activity in the heart after MI, and applied to show increased MMPs activity in infarcted region (97). An MMP-sensitive, activatable fluorescent probe (MMPSense™ 680) has been applied to visualize MMP activity in carotid plaques of symptomatic patients. Deep-tissue multispectral optoacoustic tomography (MSOT) technology visualized MMP activity in vulnerable plaques with high specificity, allowing staging of plaque vulnerability (Figures 8A,B) (15). An *ex vivo* murine MI study used a MMP imaging strategy based on activatable cell-penetrating peptide probes, and displayed high sensitivity to the proteolytic activity of MMP2 and MMP9. *Ex vivo* histology and autoradiography showed MMP activity at the infarct region and the border zone of the heart after MI (Figures 8C,D) (98).

In addition, MMP activation in atherosclerotic mouse aorta was assessed using *in vivo* SPECT/CT imaging using an ^111^In-labeled tracer targeting activated MMP (99). The study revealed the heterogeneity of atherosclerotic plaques and appeared a powerful tool for tracking plaque biology (Figure 8E). In a rabbit study, MMP activity in atherosclerotic lesions was also reported using *in vivo* SPECT imaging with a ^99^Tc-radiolabeled MMP inhibitor molecule. Tracer uptake quantification proved a feasible method for MMP detection and assessment of plaque vulnerability (100). Similarly, [^123^I]I-HO-CGS 27023A, a broad-spectrum radiolabeled MMP inhibitor, was used to image MMP presence using SPECT, at ligated carotid arteries in *apoE*^−/−^ mice (101). An ^111^In-labeled small molecule with broad specificity for activated MMPs (RP782) has been used for *in vivo* microSPECT/CT imaging of the remodeling process following mechanical injury in *apoE*^−/−^ mice (102).

Moreover, non-invasive MRI has been used to evaluate MMPs in atherosclerotic rabbits and mice using a Gd-based contrast agent. *In vivo* MRI and *ex vivo* validation demonstrated the accurate visualization and delineation of MMP-rich atherosclerotic plaques (103). Similarly, *in vivo* MR imaging of MMPs and their activity in ECM was performed in atherosclerotic rabbits using a Gd-based contrast agent. This study supported the use of MRI as a clinical tool for *in vivo* detection of vascular remodeling (104). In the thrombosis section above, we discussed a dual imaging approach that targeted not only vulnerable plaques by quantifying MMP activity with a fluorescent probe, but also endothelial injury by imaging the plaques with Gd-enhanced MRI (Figures 5A,B) (75).

#### Extracellular Matrix

The ECM provides structural support and promotes force transmission in the heart and the large vessels. The deposition and degradation of the ECM are well-orchestrated and dynamic. Accordingly, imbalanced ECM rearrangement is part of the pathogenesis of CVDs. As endothelial cells are embedded in the ECM, it is a crucial factor of endothelial biology in CVDs. In the sections above, we have already discussed some components of the ECM, including integrin, thrombotic factors and MMPs. Other targets in the cardiac and vessel wall ECM, discussed below, are collagen and elastin. Fibrosis is a series of actions through which the ECM expands. This process alters organization of collagen networks and increases levels of collagen. Fibrosis occurs either at one spot during MI or more diffusely throughout the cardiac tissue, with certain patterns correlating to different stages of heart failure (105). Thus, imaging of collagen can qualitatively and quantitatively reveal ECM rearrangements, such as fibrosis.

**Figure 9:**
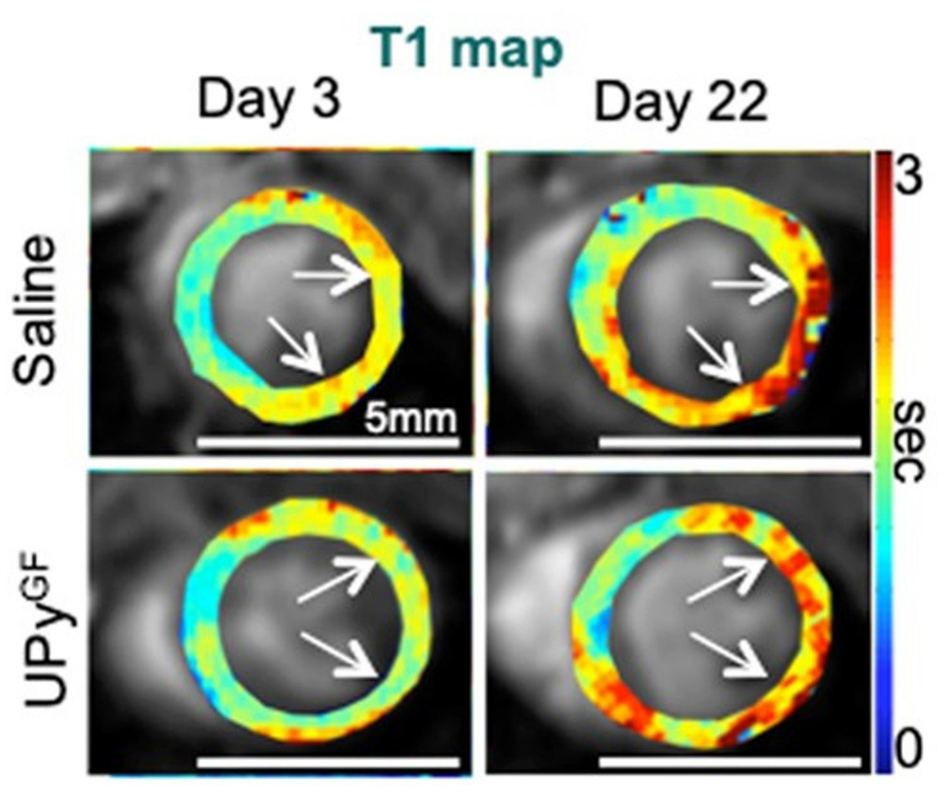
Extracellular matrix and fibrosis imaging. Longitudinal T_1_ mapping uncovers reduced fibrosis throughout cardiac healing in UPyGF-hydrogel-injected infarcts. Longitudinal T_1_ mapping of healthy hearts and hearts after I/R injected with saline or UPyGF-hydrogel [adopted from (43)].

PET and SPECT imaging methods using radioactive probes that bind to target ECM molecules have been developed. These approaches can image ECM with the highest specificity and sensitivity, but due to their low resolution, the images are often difficult to interpret. To date, some PET and SPECT probes targeting collagen, have been tested on animal models for fibrosis, but have not made their way to the clinics yet (96). Because of its high resolution, MRI has been suggested a promising technique for quantifying ECM at the heart and the large vessels *in vivo*. In our recent work, we used cardiac T_1_-mapping to measure ECM fibrosis after MI. The study measured several aspects of myocardial healing during a hydrogel-based regenerative treatment after MI. Besides the use of cardiac T_1_-mapping (Figure 9), DCE-MRI and strain imaging were performed in a single imaging session. This *in vivo* imaging platform enabled the accurate detection of improved regeneration after MI (43).

A Gd-based MRI study targeted type I collagen for fibrosis imaging, and detected elevated levels of collagen at the infarcted zone after MI (106). *In vivo* MRI-based plaque characterization by quantification of intraplaque elastin content in a murine atherosclerotic study was performed using an elastin-specific Gd-based contrast agent. Such monitoring of changes in elastin content and the high abundance of elastin during plaque development, provide a non-invasive tool for assessing plaque burden in atherosclerosis (107). Another *in vivo* MRI protocol has been established to quantify elastin content using a Gd-based elastin-binding contrast agent in a murine MI model. This ^1^H/^19^F MRI study simultaneously assessed inflammation, by perfluorocarbon nanoparticle injection that target macrophages and consequently inflammation, alongside with elastin remodeling, and identified the interplay between these two biological processes affects infarct healing (108). Thus, changes in ECM content during infarct healing and plaque development, provide potential targets for non-invasive assessment of plaque burden and cardiac outcome.

## Discussion

The development of preclinical vascular imaging techniques has provided insights into vascular biology allowing for clinical translation of these imaging techniques. Despite significant advances in vascular imaging over the past decades, some challenges remain. Nevertheless, exciting multimodal opportunities lie ahead for imaging and quantitation of vascular pathologies, possibly in conjunction with imaging of other cellular processes in CVDs, such as cardiac muscle imaging. The strength of multi-scale imaging lies in the combination of imaging probes, which enables exploitation of the advantages of each imaging modality while compensating for the disadvantages by using an additional imaging modality. Particularly for vascular pathologies, we envision that multi-scale, multimodal imaging studies can provide for early detection of key changes in endothelial cells and, in that way, report on cardiovascular risk at an early stage. Processes such as increased vascular permeability, vessel wall inflammation, expression of adhesion molecules or extracellular matrix remodeling, have the potential to serve as single or combined targets for early diagnosis of endothelial changes during the silent phase of CVDs, with possible value in predicting disease, assessing severity and quantitating the effects of therapeutic interventions.

### Basic-Science Perspective

From a basic-science perspective, it is not fully understood how early endothelial changes evolve and if there are biomarkers that can detect early events and predict adverse cardiovascular events. Probing early endothelial biomarkers or changes at the endothelial lining, may provide novel insights into endothelial cell behavior and functioning and into their interactions with immune cells. For example, imaging of interactions and regulation of Delta L4 and Notch1 signaling in CVD is largely unexplored (10).

Past preclinical vascular imaging research in CVDs has focused on vital organs, such as the heart and large vessels. However, the exploration of other vascular beds related to inflammatory processes may be the key to understanding CVDs on a system level. Recently, we used a multi-scale imaging approach to probe the hematopoietic bone marrow vasculature, the gatekeeper of massive release of innate immune cells after acute inflammation (109). *In vivo* multichannel single cell microscopy toward multiplex PET/SPECT/MRI studies are expected to drive discovery of molecular and cellular interactions in different organ systems in CVD.

Of course, developing novel imaging techniques should be accompanied by more comprehensive biological profiling, including flow cytometric analysis of endothelial cells and other biological assays. Another critical need is higher-sensitivity imaging that will enable the visualization of molecular changes in endothelial cells during disease progression. Even though optical methods are certainly capable of following endothelial cell changes, they are difficult to implement in the deep-cardiac and large-vessel arenas. There is still a lack of preclinical imaging methods capable of tracking molecular changes at high resolution in deep tissue and throughout the whole body. More recently developed imaging methods, such as photoacoustic imaging, might reach such sensitivity levels.

### Clinical Considerations

Multi-scale imaging of vascular pathologies can potentially advance cardiovascular medicine in multiple ways. Because molecular-imaging-based strategies can provide insights into biological processes in vascular phenotypes, they can improve risk assessment and early detection of disease. Such developments can eventually lead to improved personalized medicine and better monitoring of therapeutic efficacy and outcome. Moreover, experimental multi-scale imaging will improve our basic biological understanding of vascular pathologies, and subsequently set the stage for new routes for diagnostic and therapeutic developments.

Clinically established imaging of vascular pathologies includes structural imaging, such as echocardiography and cardiac MRI (27), contrast-enhanced ultrasound imaging to measure microvascular blood flow and blood volume (23), as well as targeted dual-isotope PET viability studies with ^13^N-ammonia and ^18^F-FDG, to measure myocardial perfusion and viability (20, 24, 26, 27). Other examples of clinically targeted vascular imaging include imaging of vascular permeability of carotid arteries (38, 110), imaging of vessel inflammation by iron oxide nanoparticles (59), PET/MRI to detect α_*v*_β_3_ integrin activation and predict outcome in infarct patients (89) and magnetization transfer MRI for fibrosis evaluation (111).

With the development of gene or stem cell therapy for CVD, costs, and complexity of clinical development are continuing to rise. Multi-scale molecular imaging could help identify promising therapies at an early stage of development of a potentially new therapy, reducing these escalating costs. For example, we recently described a multiparametric MRI approach, that gave insights into various biological aspects of a novel treatment for ischemic heart disease (i.e., vascular permeability, myocardial strain, and extracellular matrix remodeling) (43). The classical structural evaluation of anatomical changes to assess clinical outcomes in CVD tends to ignore delicate key biological changes. Therefore, complex new therapeutic strategies will necessitate advanced multi-scale vascular imaging for a more sensitive and multi-marker assessment of their therapeutic efficacy and safety, expected to shorten the therapy development process.

### Summary

This review attempted to clarify, illustrate, and expound on multi-scale imaging technologies applied to visualize endothelial cell alterations appearing during the pathogenesis of CVDs. Spatially and temporally resolved molecular imaging of endothelial changes is expanding our understanding of how endothelial cells change and interact during the progression of CVDs. The non-invasive assessment of these processes and the integration of multi-scale technologies, represent an extraordinary opportunity to decode the cellular and molecular activities of endothelial cells at unparalleled resolution. These developments in preclinical vascular imaging science are expected to help drive a new era of precision medicine in the treatment of CVDs. Although, CVDs are highly complex involving multiple cell types, systemic changes and biological mechanisms driving disease progression, we believe that multifaceted vascular imaging approaches could pave the way toward a better understanding of endothelial involvement in CVDs health in the future.

## Author Contributions

AT and KV reviewed and synthesized the relevant information and were the primary authors of this review article. GS and BE made substantial contributions to the drafting and revision of the article. All authors contributed to the article and approved the submitted version.

## Funding

AT was supported in part by a grant from the Zeff Fellowship. KV was supported by the Israeli Science Foundation 446/21 and 660/21.

## Conflict of Interest

The authors declare that the research was conducted in the absence of any commercial or financial relationships that could be construed as a potential conflict of interest.

## Publisher's Note

All claims expressed in this article are solely those of the authors and do not necessarily represent those of their affiliated organizations, or those of the publisher, the editors and the reviewers. Any product that may be evaluated in this article, or claim that may be made by its manufacturer, is not guaranteed or endorsed by the publisher.
